# Effect of Complexes of Zinc, Cobalt and Copper With D-Aminosugars on the Replication of Herpes Simplex Virus Type 1 (HSV-1)

**DOI:** 10.1155/MBD.1997.35

**Published:** 1997

**Authors:** S. Shishkov, T. Varadinova, M. Panteva, P. Bontchev

**Affiliations:** 1 Laboratory of Virology Faculty of Biology Sofia 1421 Bulgaria; 2 Faculty of Chemistry Sofia University 8 Dragan Tzankov Blvd. Sofia 1421 Bulgaria

## Abstract

Our previous results show that Zn(pic)_2_ and Zn(asp)_2_ inhibit key steps of the replication of HSV-1. Anti-HSV effect of complexes of Co(II) with aminoacids Lys and Ser was also found. In the present study we describe the effect of complexes of Zn(II), Co(II) and Cu(II) with D-aminosugars on the replication of HSV-1 and on the infectivity of free virions. The experiments were done using primary rabbit kidney cells (r.k.), diploid human embryonal fibroblasts (F) and Vero cells. No differences in the toxicity of metal complexes on diploid cells- r.k. and F, were found. Neither metal complexes, nor ligands-galactosoxime and glucosoxime, influenced the viral replication. During 1-4h prolonged contact only Cu(Gl.NOH)_2_ inactivated HSV-1 virions up to 90%. The results show that D-aminosugars are not suitable ligands for Zn(II), Cu(II) and Co(II) in respect of the inhibition of viral replication. However, only Cu(Gl.NOH)_2_ was able to inhibit the infectivity of free virions.


### EFFECT OF COMPLEXES OF ZINC, COBALT AND COPPER WITH D-AMINOSUGARS ON THE REPLICATION OF HERPES SIMPLEX VIRUS TYPE 1 (HSV-1)

S. Shishkov[1], T. Varadinova[1*], M. Panteva[1] and P. Bontchev[2]

[1] Laboratory of Virology, Faculty of Biology and [2] Faculty of Chemistry, Sofia University, 8 Dragan Tzankov Blvd., 1421 Sofia, Bulgaria

### Abstract

Our previous results show that $Zn(pic)_2$ and $Zn(asp)_2$ inhibit key steps of the replication of HSV-1. Anti-HSV effect of complexes of Co(II) with aminoacids Lys and Ser was also found. In the present study we describe the effect of complexes of Zn(II), Co(II) and Cu(II) with D-aminosugars on the replication of HSV-1 and on the infectivity of free virions. The experiments were done using primary rabbit kidney cells (r.k.), diploid human embryonal fibroblasts (F) and Vero cells. No differences in the toxicity of metal complexes on diploid cells - r.k. and F, were found. Neither metal complexes, nor ligands - galactosoxime and glucosoxime, influenced the viral replication. During 1- 4h prolonged contact only $Cu(Gl.NOH)_2$ inactivated HSV-1 virions up to 90%. The results show that D-aminosugars are not suitable ligands for Zn(II), Cu(II) and Co(II) in respect of the inhibition of viral replication. However, only $Cu(Gl.NOH)_2$ was able to inhibit the infectivity of free virions.

**Key words:** Herpes simplex virus type 1, D-aminosugars, inhibition, zinc, cobalt, copper

### Introduction

Among metals essential for life and according to the amount in the human body, zinc is the second most abundant trace metal after the iron (1-4). The physiological functions of zinc are primarily related to its presence in the active site of up to 200 metalloenzymes (5). Apart from the presence of cobalt as a constituent of vitamin $B_{12}$, this metal also influences many other physiological and enzymatic functions (1-3, 6). The copper, being an essential trace metal, is a part of the active site of at least 60 metalloenzymes (2, 3, 7).

A specific compartment within the cell predetermines the activity of the particular metal ion. Thus, zinc acts mainly in the cytoplasm. Small amounts of zinc participate also in the nucleus as a constituent of proteins involved in the regulation and the expression of the genome (2,3). Cobalt realises its activity only in the cytoplasm, while copper acts in extracellular space (3).

Moreover, during the regulation of a wide range of metabolic processes these metals interact with each other and their main characteristic, as well as that of their compounds, is their extremely low toxicity for humans (6, 8).

Because of the significant role of the ligand in the expression of the activity of a particular metal ion, we decided to study the effect of aminoderivatives of D-glucose and D-galactose, as well as of their complexes with Zn(II), Cu(II) and Co(II) on HSV-1 infection in cell cultures. These experiments are a part of a large investigation on the role of complexes of the above metal ions with different bioligands on HSV-1 replication (9-11).

### Materials and Methods

***Virus and cells.*** HSV-1, strain Victoria; primary rabbit kidney cells (r.k.), diploid human embryonal fibroblasts (F), and Vero cells were used in the experiments.

***Ligands.*** Aminoderivatives of D-glucose - glucosoxime (Gl.NOH), and of D-galactose - galactosoxime (Ga.NOH), were used as ligands of Zn(II), Co(II) and Cu(II).

***Maximal nontoxic concentration (MNC).*** Cells from monolayers were washed and covered with media modified with different concentrations of metal complexes or ligands. Microscopically, degeneration of monolayers and changes of cell morphology were investigated from the 24th till the 96th h. Samples of cells grown in nonmodified medium served as a control. Each experiment was duplicated. The highest concentration in the presence of which and during the whole period of investigation the morphology of the cells and monolayers was similar to that in the control, is recognised as MNC.

***Infectious virus titre.*** Cells from confluent monolayers grown in 96 well plates were infected with ten-fold dilutions of HSV-1. Infectious virus titre was determined at the 48th h after culturing at 37_C by Reed and Muench (12).

***Effect of metal complexes on the replication of HSV-1.*** Experiments were done in multicycle growth conditions. Confluent cell monolayers from 96 well plates were washed and infected with ten-fold dilutions of HSV-1. After 1h for adsorption, cells were covered with media modified with different concentrations of metal complexes and of ligands. One set of infected cells served as untreated control. The effect on the replication of HSV-1 was determined at the 48th h after culturing at 37_C by reduction of infectious virus titres as compared to that in the control.

***Effect of metal complexes on extracellular (free) HSV-1 virions - virucidal effect.*** Equal volumes of HSV-1 stock containing 100 pfu/0.1 ml and medium modified with the appropriate metal complex or the ligand, in MNC, were incubated at 37_C for 15min, 30min, 1h, 2h, 4h , 6h and 12h. The infectious titre of each sample was determined on the 48th h. Data were compared to those of viral control - equal volumes of HSV-1 stock and nonmodified medium incubated as described above.

### Results

The data presented in table 1. show that the metal complexes are 1000 times more toxic than the ligand. It is interesting to note a specific response of Vero cells after the action with Co(II) complexes. Thus, $Co(Ga.NOH)_2$ is 10 times more toxic (MNC 0,01 μM) than $Co(Gl.NOH)_2$ (MNC 0,1 μM).

Table 1. Summarised data on the toxicity of complexes of Zn(II), Co(II) and Cu(II) and of D-aminosugars in r.k., F and Vero cells.

| Action with: | Concentration, μM | | | | |
|---|---|---|---|---|---|
| | 100 | 10 | 1 | 0.1 | 0.01 |
| Ga.NOH | + | - | - | - | - |
| $Cu(Ga.NOH)_2$ | + | + | + | - | - |
| $Co(Ga.NOH)_2$ | + | + | + | - (+)* | - |
| $Zn(Ga.NOH)_2$ | + | + | + | - | - |
| Gl.NOH | + | - | - | - | - |
| $Cu(Gl.NOH)_2$ | + | + | + | - | - |
| $Co(Gl.NOH)_2$ | + | + | + | - | - |
| $Zn(Gl.NOH)_2$ | + | + | + | - | - |

(+)* - toxic concentration for Vero cells.

No differences in the toxicity of the metal complexes on diploid cells - r.k. and F, were found. In order to obtain comparative data, experiments on the effect of metal complexes and ligands on HSV-1 infection were done on diploid cells.

Neither metal complexes, nor ligands - Ga.NOH and Gl.NOH, influenced the replication of HSV-1 in r.k. and in F cells (data not shown).

The only complex able to inactivate free HSV-1 virions, but not viral replication, is $Cu(Gl.NOH)_2$ (table 2 and fig. 1). During 1-4 h prolonged contact 90% of HSV-1 virions were already inactivated. Contrary, complexes of Zn(II) and Co(II), as well as $Cu(Ga.NOH)_2$ had no effect on free HSV-1 virions (table 2).

### Discussion

In 1970 Spear et al. (13,14) have shown that within 4-5 h after HSV infection the only apparent protein glycosilation taking place in the infected cells is that of virus-specific proteins. The result is the deposition in infected cell membranes of virus-specific glycoproteins. On the other hand, in 1976 Brennan et al. (15) have reported that HSV glycoproteins presented in the infected cells are not suitable substrates for the attachment of glucose units. They suggest that in HSV infected cells enhanced glycolysis or abnormal compartmentalisation of nucleotide sugar pools is probably involved. On the other hand, 2-deoxy-D-glucose interfere with the synthesis of HSV glycoproteins. As a result, production of infectious virus progeny and the fusion between cells are markedly inhibited (12,16-19).

Table 2. Effect of ligands and of their complexes with Zn(II), Co(II) and Cu(II) on extracellular HSV-1 virions*.

| Action with♦ | Duration of the contact | | | | | | |
|---|---|---|---|---|---|---|---|
| | 15 min | 30 min | 1 h | 2 h | 4 h | 6 h | 12 h |
| Ga.NOH | 6.5 | 6.3 | 6.5 | 6.5 | 6.3 | 5.5 | 4.5 |
| Cu(Ga.NOH)$_2$ | 6.5 | 6.5 | 6.5 | 6.6 | 6.5 | 5.5 | 4.5 |
| Co(Ga.NOH)$_2$ | 6.5 | 6.5 | 6.3 | 6.5 | 6.5 | 6.0 | 4.5 |
| Zn(Ga.NOH)$_2$ | 6.5 | 6.5 | 6.5 | 6.5 | 6.5 | 5.5 | 4.5 |
| Gl.NOH | 6.5 | 6.5 | 6.3 | 6.5 | 6.3 | 5.5 | 4.5 |
| Cu(Gl.NOH)$_2$ | 6.3 | 6.3 | 5.5 | 5.5 | 5.5 | 5.3 | 4.5 |
| Co(Gl.NOH)$_2$ | 6.2 | 6.5 | 6.5 | 6.5 | 6.0 | 5.5 | 4.5 |
| Zn(Gl.NOH)$_2$ | 6.5 | 6.5 | 6.5 | 6.5 | 6.5 | 5.5 | 4.3 |
| HSV-1 | 6.5 | 6.5 | 6.5 | 6.5 | 6.5 | 5.5 | 4.5 |

*- data are presented in $\log_{10}$ pfu/0.1 ml. ♦- compounds are applied in MNC (see tabl. 1)

The present data show that aminoderivatives of D-glucose (Gl.NOH) and of D-galactose (Ga.NOH) are 1000 times less toxic than the appropriate metal complex - MNC 10 μM. There is no doubt that the low toxicity of Gl.NOH and of Ga.NOH is due to the fact that these sugars, being natural products, directly participate in the normal cell metabolic pathways and must be well tolerated by cells in vitro as well. The higher toxicity of metal complexes MNC 0,1μM, as compared to that of the appropriate ligand, is due to the presence of the metal ions.

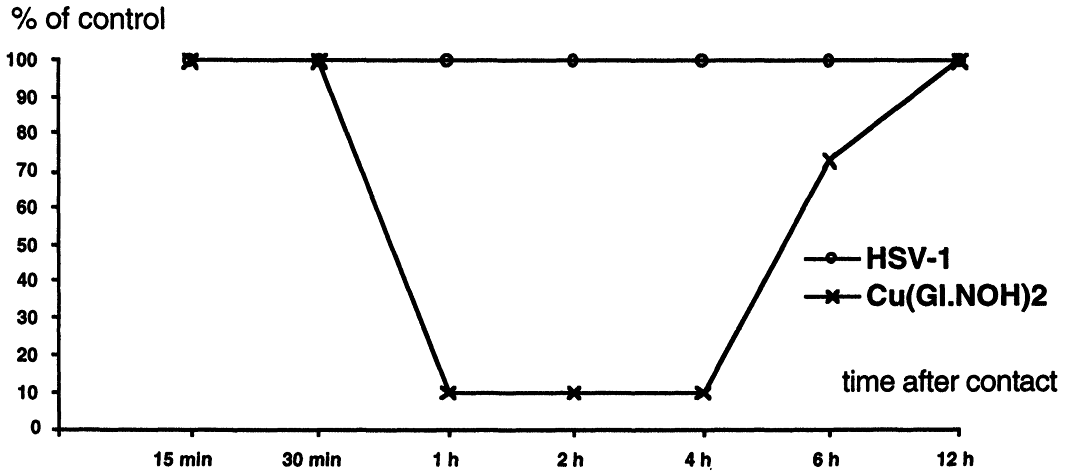

Fig. 1. Virucidal effect of Cu(Gl.NOH)$_2$

Nevertheless, these complexes cannot interfere with the production of HSV-1 progeny. The only inhibitory effect of Cu(II) bound to Gl.NOH - Cu(Gl.NOH)$_2$ ,but not to Ga.NOH, on the infectivity of extracellular virions was found. This is probably due to the fact that Cu(Gl.NOH)$_2$ is localised in the extracellular space, a compartment specific for Cu(II), thus inhibiting the fusion between viral envelope and cell membrane, similarly to the inhibition of cell fusion obtained by Brennan et al. (15) after action with 2-deoxy-D-glucose.

On the bases on the present and of previously published data, we can conclude that the anti-HSV effect of the particular metal ion strongly depends on the ligand bound to it. Thus, the aminoderivatives of D-glucose and D-galactose are not suitable ligands for Zn(II), Cu(II) and Co(II) in respect to the inhibition of HSV-replication. Furthermore, suitable ligands for Cu(II) and Co(II) in respect to the virucidal effect of the particular complex are Gl.NOH and serine (11). However, in order to inhibit HSV-1 replication within the host cell, complexes of

Zn(II) with picolinic and with aspartic acids are recommended (9). In addition, the latter complexes posses virucidal activity towards VZV virions, which is due to the virus-specific properties (11).

**Acknowledgements**
This work was partially supported by grants L-451 and X-405 from the National Scientific Foundation.

**References**

1. M.P.Fletcher, M.E.Gerswin, L.Hurley. *In: Tumor Immunology and Immunoregulation by Thymic Hormones*. F.Dammaco (ed.)., Masson S.p.A., Milan, 1987, 57.
2. *The Biological Chemistry of the Elements. The Inorganic Chemistry of Life*. J.J.R.Da Silva, R.J.P. Williams (eds.)., Claredon Press, Oxford, 1993, 299.
3. *Comprehensive Co-ordination Chemistry*. Sir G. Wilkinson (ed.)., Pergamon Press, New York, 1987, Vol.**5**, 925.
4. R.L.Berthold. *Zinc. In: Handbook on Toxicity of Inorganic Compounds*. H.G.Seiler, H.Sigel (eds.)., Marcel Dekker Inc., New York, Basel, 1988, 787.
5. A.S. Prasad. *CRC Critical Rev. Clin. Lab. Sci.*, 1977, **8**,1.
6. J. Angere, R. Heinrich. *Cobalt. In: Handbook on Toxicity of Inorganic Compounds*. H.G.Seiler, H.Sigel (eds.)., Marcel Dekker Inc., New York, Basel, 1988, 251.
7. B. Sarkar. *Copper. In: Handbook on Toxicity of Inorganic Compounds*. H.G.Seiler, H.Sigel (eds.)., Marcel Dekker Inc., New York, Basel, 1988, 266.
8. National Research Council, *Subcommittee on zinc.*, "Zinc"., University Park Press, Baltimore, 1979.
9. T. Varadinova, S. Shishkov, P. Bontchev et al. *J. Chemother.*,1993, vol. **5**, n. 1, 3.
10. T. Varadinova, S. Shishkov, M. Panteva, P. Bontchev. *MBD*, 1996, vol.**3**, n. 3, 149.
11. S. Shishkov, T. Varadinova, P. Bontchev, C. Nachev, E. Michailova. *MBD*, 1996, vol.**3**, n. 1, 11.
12. L. Reed, H. Muench. *An. J. Hyg.*, 1938, **21**, 493.
13. P.G. Spear, J.M. Keller, B. Roizman. *J. Virol.*, 1970, **5**, 123.
14. P.G. Spear, B. Roizman. *Proc. Natl. Acad. Sci.*, U.S.A., 1970, **66**, 730.
15. P.J. Brennan, S.N. Steiner, R.J. Courtney, J. Skelly. *Virology*, 1976, **69**, 216.
16. H. Ludwig, H. Becht, R. Rott. *J. Virol.*, 1974, **14**, 307.
17. H. Ludwig, R. Rott. *J. Virol.*, 1975, **16**, 217.
18. G. Kaluza, M.F.G. Schmidt, C. Scholtissek. *Virology*, 1973, **54**, 179.
19. S. Oloffson, E. Lycke. *Arch. Virol.*, 1980, **65**, 201.

**Received: September 19, 1996 - Accepted: October 16, 1996 - Received in revised camera-ready format: February 3, 1997**

